# Knowledge and practices regarding tuberculosis infection control among nurses in Ibadan, south-west Nigeria: a cross-sectional study

**DOI:** 10.1186/s12913-020-05156-y

**Published:** 2020-04-06

**Authors:** Patrick Aboh Akande

**Affiliations:** 1 Department of Public Health, School of Health Care Sciences, Sefako Makgatho Health Sciences University, Pretoria, South Africa; 2 APIN Public Health Initiatives, Abuja, Nigeria

**Keywords:** Tuberculosis, Infection, Control, Knowledge, Practice, Nurses

## Abstract

**Background:**

Nurses are particularly vulnerable to nosocomial tuberculosis (TB) infection because, being in the frontline of healthcare provision, they are frequently exposed to patients with infectious TB disease. Although cost-effective measures are available for TB infection control (TBIC), they are often poorly implemented. Knowledge of TBIC is known to positively influence the practice of the measures. There is, however, paucity of data on the knowledge and practices regarding TBIC among nurses in Nigeria. This study was aimed at determining the levels of TBIC-related knowledge and practices of nurses in Ibadan, and their associated socio-demographic factors.

**Methods:**

This cross-sectional study utilized a self-administered questionnaire to collect data from 200 nurses in two secondary health facilities, in May 2014. The mean knowledge and practice scores of the nurses were determined and logistic regression was utilized to explore the association between the scores and socio-demographic characteristics.

**Results:**

The respondents had mean knowledge and practice scores of 68.2 and 79.9% respectively. Using cut-off points of 80 and 100% for good knowledge and practice scores respectively, small proportions of the nurses had good scores – knowledge (10.5%) and practice (6%). Knowledge was not significantly associated with the socio-demographic characteristics of the nurses. Work experience was the only factor that was significantly associated with practices, with the more experienced nurses (> 18 years of work experience) having lower odds of obtaining good practice scores (OR 0.25, 95% CI 0.06–0.94). There was also no significant association between knowledge and practice scores (the nurses were yet to be trained on the newly-introduced TBIC package at the time of the study).

**Conclusions:**

The study revealed that small proportions of the nurses had good knowledge and practice scores. Its findings will be useful for the designing of interventions to improve TBIC among nurses and other healthcare workers, and to benchmark evaluation of the interventions. It is recommended that nurses should be trained on TBIC to equip them with necessary knowledge and skills. This, together with appropriate policy directives, and adequate monitoring and supervision will contribute to optimal implementation of TB preventive measures.

## Background

Tuberculosis (TB) is a major global public health problem. It is caused by a microorganism called *Mycobacterium tuberculosis* (MTB) and commonly affects the lungs (pulmonary TB or PTB) and this accounts for about 85% of all TB cases [1]. TB can also affect other organs in the body (extrapulmonary TB): lymph nodes, abdomen, bones and joints, pericardium, pleura, genitourinary system and meninges; and it can also be generalized. PTB is the most important source of TB transmission as MTB is carried in air-borne droplets or aerosols produced when a person infected with PTB coughs, sneezes, spits, talks or sings. TB is preventable and there is affordable and effective treatment for it. In 2017, an estimated 10 million new cases of TB were recorded globally, with the African region accounting for 25% of these cases; and worldwide, TB was the leading cause of death due to an infectious disease [2].

With a 2017 estimated population of 182 million people, Nigeria is the most populous country in Africa [2]. It ranks sixth among the countries with the highest TB burden in the world and is included in the three lists of 30 high burden countries (HBC) compiled by World Health Organization (WHO) for TB, TB/human immunodeficiency virus (TB/HIV), and multi-drug resistant TB (MDR-TB); for the period 2016–2020. Each of these lists accounts for about 90% of the global burden [2]. Nigeria is one of the 14 countries that appeared in all the three lists. It reported 418,000 incident TB cases in 2017 (incidence rate: 219/100,000 population). TB was successfully treated in 86% of all cases registered in the country in 2016, and approximately 155,000 people died from it in 2017. The country has an MDR-TB/RR-TB prevalence of 4.3 and 25% among new cases and previously treated cases respectively [2].

Nosocomial TB transmission, which means the spread of TB in health facilities, is an enormous challenge for healthcare workers (HCWs) worldwide. The increased risk of nosocomial TB transmission among HCWs has been well-documented and HCWs have a higher incidence of TB disease than the general population [3–6]. The increased exposure of HCWs to infectious TB patients, especially where there is inadequate implementation of TB infection control (TBIC) measures, accounts for the heightened risk [7–9]. Strict adherence to recommended control measures is required for effective TBIC. WHO has recommended the adoption of several TBIC measures because of the diverse nature of the risk factors for TB transmission in health facilities [10]. These measures include (i) managerial measures; (ii) administrative measures – which are considered the first priority even in resource-limited settings;(iii) environmental control measures; and (iv) personal protection equipment (PPE) e.g. particulate respirator. Low- and medium-income countries (LMIC) can adopt simple, practical and cost-effective interventions to reduce the exposure of HCWs to infectious TB patients [11]. TBIC measures have been successfully implemented in most high-income countries and in some resource-limited settings [12]. However, poor implementation of the control measures by HCWs has been reported [13–15]. Good knowledge of occupational TB exposure is known to positively influence the adoption of TBIC practices by HCWs [16, 17]. HCWs have, however, been shown to have varying levels of knowledge and practices regarding TBIC and a good understanding of TBIC does not necessarily equate to adequate practice of the measures [9, 18]. In addition to poor knowledge, identified barriers to effective implementation of TBIC include weak managerial support, poor funding, limited work space and inadequate staffing [19].

The results of surveys conducted in Nigeria to assess the level of implementation of TBIC practices in health facilities have generally been poor [14, 18, 19]. National guidelines for the implementation of TBIC had been rolled out at the time this study was undertaken [20]. However, the policy had not fully trickled down to lower healthcare levels. Moreover, training on the guidelines was yet to be widespread and most facilities providing care for TB patients were still putting administrative control measures in place [14, 18, 19]. Nursing staff are at great risk of acquiring TB because they are in the frontline of healthcare provision and are frequently exposed to patients with infectious TB disease [21, 22]. There is, however, paucity of data on TBIC-related knowledge and practices among nurses in Nigeria. Available studies that address individual HCWs are either focused on personnel in specific units such as TB clinics or diffusely include general HCWs [23, 24]. In spite of this, the results of these studies show gaps in knowledge and practice of the measures. Further studies that specifically target nurses are needed since they usually have the earliest contact with infectious TB cases in facilities and can, therefore, play a crucial role in controlling the spread of TB.

Previous studies have explored the influence of HCWs’ socio-demographic characteristics on their knowledge and practice of TBIC measures. Researchers in Addis Ababa, Ethiopia, found TBIC knowledge to be significantly associated with work experience [25]. On the other hand, studies in Northwest Ethiopia and Uganda did not show any significant association between it and age or work experience [26, 27]. Regarding the practice of TBIC measures, investigators in Lesotho and Ethiopia observed that this was not associated with age, work experience and marital status of HCWs [9, 25, 28]. However, TBIC practice was reported to be significantly related to its knowledge in studies in South Africa and Ethiopia [15, 26].

The aim of this study was to determine the levels of TBIC-related knowledge and practices of nurses in Ibadan, South-West Nigeria, and their associated socio-demographic factors. The study outcomes will contribute to the general body of knowledge on the status of implementation of TBIC measures and will provide useful baseline information for the designing of appropriate interventions to address issues related to this and for subsequent evaluation of the interventions.

## Methods

### Study design and setting

This cross-sectional study was conducted in Ibadan, Oyo State, located in South-West Nigeria. With a 2011 population of about 3,034,206 (density of 985/km^2^), Ibadan is the third largest metropolitan area in Nigeria, and the largest by geographical area (3080 km^2^) [29, 30]. Oyo State reported 6901 TB cases in 2017, making it the state with the third highest burden in Nigeria [31].

### Study population and sample

The study population consisted of nurses who work at two secondary health facilities in Ibadan metropolis – Ring Road State Hospital and Adeoyo Maternity Teaching Hospital, located in two separate Local Government Areas (Ibadan South-West and Ibadan North respectively). The study sites were purposively selected. Based on available administrative data, 173 and 217 nurses respectively at these facilities made up the study population (total = 390). Yamane’s approach was used to determine the sample size: *n = N/(1 + Ne*^*2*^*)*, where *N* is the population size and *e* is the margin of error (taken as 5%) [32]. A sample size of at least 198 nurses was determined. To compensate for non-response, this was increased by 10% to 218. The facilities were proportionately allocated sample sizes of 96 and 123 respectively (both approximated to the nearest decimal). However, all available nurses at the study sites were encouraged to participate because of the public health and health system benefits of the study.

### Study instrument

The study instrument was a self-administered questionnaire that had a section on socio-demographic characteristics, as well as scales on TBIC-related knowledge and practices [Additional file 1]. The scales were adapted from an instrument previously utilized to study TBIC in a high drug-resistance setting in South Africa by Kanjee et al. [13]. Subject matter experts in Nigeria (two consultant chest physicians and two senior TB nurses) examined the instrument for face validity to ensure that the items were relevant, adequate and appropriate. The review of the instrument undertaken by the Sefako Makgatho Health Sciences University Research Ethics Committee served to improve its content validity. Some of the initial instrument items were deleted and others rephrased based on input by the experts. The knowledge and practice scales had acceptable internal consistency, with Cronbach’s alpha values of 0.6 and 0.8 respectively. Fifteen nurses, who were eventually excluded from the main study, participated in the pilot-testing of the questionnaire at one of the study sites. Their understanding of the questions and the challenges encountered in responding to them informed the rephrasing of some of the items for clarity. The study instrument was drafted in English and there was no need for translation as this is the official language in the workplace and the medium of instruction for nursing education in Nigeria. The final knowledge scale contained 33 items, with each having response options of “true”, “false”, or “I don’t know”. Each correct answer had a score of “1” and an incorrect answer “0”, while “I don’t know” was considered an incorrect answer. The scale had a maximum possible score of 33. The practice scale had six items which measured self-reported frequency of adherence to various practices. It was scored using a 5-point Likert-type scale: “never” (1 point), “rarely” (2), “sometimes” (3), “often” (4), and “always” (5); and it had a maximum possible score of 30.

### Data collection

Two research assistants and a supervisor were recruited and trained for the study. The nurses were informed about the study through their administrative structure. After explaining the purpose of the study to the participants, each of them that agreed to take part in the study received a copy of the information leaflet, consent form and study questionnaire. After signing the consent form, the questionnaire was issued out and this was collected after completion by the nurses. The study was conducted in May 2014.

### Statistical analysis

STATA version 13 (StataCorp, College Station, TX) was used to analyze the study data. The socio-demographic variables of the nurses and their levels of knowledge and practice were described using means for continuous variables and frequency for categorical ones. The knowledge score of each nurse was presented as a percentage of the maximum possible knowledge score i.e. score obtained/33 × 100%. Similarly, the practice score was given as a percentage of the maximum possible practice score: score obtained/30 × 100%. In addition, both scores were categorized into good and poor scores. Scores of 80% and above on the knowledge scale were regarded as good scores while those below 80% were taken as poor scores. Regarding the practice scale, scores of 100% were taken as good scores while those less than this value were poor scores. The cut-off point for good practice score was fixed at 100% because optimal performance of TBIC measures is essential to minimize the nurses’ risk of contracting TB. Binomial logistic regression was used to determine associations between socio-demographic dichotomous categories as independent variables, and knowledge and practice as separate dependent variables (both having dichotomized outcomes of “good” and “poor” scores); as well as between their knowledge (independent variable) and practice (dependent variable). The socio-demographic factors examined were categorized as follows for the purpose of the logistic regression analysis: age (using the median age of 44 years as cut-off point to give **= <** 44 and > 44 years); work experience (**= <** 18 and > 18 years, with the median age of 18 years as cut-off); sex; and marital status (married and unmarried). Professional rank was also grouped into senior category (Principal Nursing Officer, Assistant Chief Nursing Officer and Chief Nursing Officer) and junior (Nursing Officer and Senior Nursing Officer). The level of statistical significance was set at *p* < 0.05.

### Ethical considerations

The study was approved by Sefako Makgatho Health Sciences University Research Ethics Committee (MREC/H/271/2013: PG) and Oyo State Ministry of Health Research Ethical Review Committee in Nigeria (AD 13/479/557). Permission was obtained from Oyo State Hospitals Management Board and the management of both study sites. Participation in the study was completely voluntary and measures were taken to ensure privacy and confidentiality of the participants. Informed consent was obtained from each participant and their names, addresses and other unique identifiers were left out of the questionnaire for the sake of anonymity. Individual participant questionnaires and the facilities were allocated identification numbers, which could not be linked to the participants.

## Results

At the facility with the larger sample size, 100 nurses out of the required 123 returned their completed questionnaires. An additional four nurses voluntarily took part in the study at the facility that had an initial sample size of 96 allocated to it, bringing the number of respondents there to 100. Altogether, completed questionnaires were obtained from 200 respondents out of the sample size of 218 (response rate = 92%).

### Socio-demographic characteristics of the respondents

The mean age of the nurses was 43.7 years, as shown in Table 1. They also had a mean work experience of 19.3 years. The vast majority of them were females (97.0%) and married (91.5%). More of them were also in the senior professional category (59%).

**Table 1:** Socio-demographic characteristics of the respondents (*N* = 200)

Variable	Mean (SD), years	*n* (%)
Age	43.7 (8.98)	
Work Experience	19.3 (9.72)	
Sex		
Female		184 (97.0)
Male		6 (3.0)
Age category		
= < 44 years		102 (51.0)
> 44 years		98 (49.0)
Work experience category		
= < 18 years		101 (50.5)
> 18 years		99 (49.5)
Professional rank		
Junior category		82 (41.0)
Senior category		118 (59.0)
Marital status		
Married		183 (91.5)
Unmarried		17 (8.5)

### TBIC knowledge and practice scores of the respondents

Table 2 shows the distribution of the nurses according to correct responses given to the items on the knowledge scale. The scale had three domains: TB symptoms (10 items), mode of spread and risk of TB (13 items), and TBIC measures (10 items). On the items related to TB symptoms, more than 80% of the nurses provided correct answers for the five common symptoms: cough of more than 2–3 weeks duration was correctly identified by nearly all of them (99.5%), followed by coughing up blood – 98.5%, weight loss – 97.5%, night sweats – 95.5%, and fever – 88%. Symptoms that are not related to TB were answered correctly by smaller proportions of the respondents. For example, only 45% of them correctly noted that watery eyes are not a symptom of TB. Five of the 13 items in the domain on the “mode of spread and risk of TB” were correctly answered by more than 80% of the nurses. The remaining eight items had smaller proportions with correct responses, the lowest being “Healthcare workers in the out-patients clinic have the same risk of getting TB as any other person” (7.5%). The items in this domain related to the risk of contracting TB generally had more incorrect responses, apart from “Patients with TB disease are more likely to infect others if they cough up a lot of sputum”, which had 91% of the nurses giving the correct answer. On the HIV-related items, 67.0% of the participants responded correctly to whether it is alright for HIV-positive staff who are healthy to work in TB high-risk areas of the hospital. The other two items related to increased risk for HIV-positive persons had poor responses (23 and 21.5% respectively). Out of the 10 items on TBIC measures, only three had at least 80% of the nurses providing correct responses. These included two items on cough hygiene and keeping windows open in a room with a coughing patient.

**Table 2:** Distribution of participants with correct responses (*N* = 200)

SN	Knowledge item	*n*	%
*Symptoms of PTB*			
1	Blurry vision (False)	108	54.0
2	Coughing for longer than 2–3 weeks (True)	199	99.5
3	Coughing up blood (True)	197	98.5
4	Ear pain (False)	106	53.0
5	Fever (True)	176	88.0
6	Memory loss (False)	118	59.0
7	Night sweats (True)	191	95.5
8	Pain with urination (False)	153	76.5
9	Watery eyes (False)	90	45.0
10	Weight loss (True)	195	97.5
*Mode of spread and risk of TB*			
11	TB can be spread to others through semen or vaginal fluid (False)	175	87.5
12	TB can be spread to others through the air (True)	193	96.5
13	TB can be spread to others through contact with blood (False)	153	76.5
14	Patients with TB disease can infect other people by coughing (True)	193	96.5
15	Patients with TB disease can infect other people by sharing food (False)	87	43.5
16	Patients with TB disease can infect other people by talking or singing (True)	90	45.0
17	Patients with TB disease can infect other people by sneezing (True)	178	89.0
18	Patients with TB disease are more likely to infect others if they cough up a lot of sputum (True)	182	91.0
19	Treating TB patient with the right drugs does not affect how infectious they are (False)	90	45.0
20	Healthcare workers in the outpatients clinic have the same risk of getting TB as any other person (False)	15	7.5
21	An HIV-positive person has the same risk of getting TB as an HIV-negative person (False)	46	23.0
22	An HIV-positive staff member cannot get sick with TB if they practise TB infection control measures (False)	43	21.5
23	It is alright for HIV-positive staff who are healthy to work in TB high risk areas of the hospital (False)	134	67.0
*TB infection control (TBIC) measures*			
24	When entering the outpatients clinic, every patient should be asked if they are coughing (True)	136	68.0
25	Patients who are identified as presumptive TB cases should not be separated from other patients in the waiting area as this will be seen to be discriminating against them (False)	117	58.5
26	A coughing/sneezing patient should be instructed to cover their mouth with a handkerchief, tissue or their arm while coughing/sneezing (True)	192	96.0
27	If coughing/sneezing TB patients or presumptive cases use handkerchief or tissue to cover their mouth while coughing or sneezing, that is usually enough to protect the healthcare worker (False)	97	48.5
28	If a coughing patient has not been diagnosed as a case of TB, it is not necessary to instruct them to cover their mouth while coughing (False)	173	86.5
29	A coughing patient should be instructed to collect a sputum sample in the clinic toilet (False)	79	39.5
30	Opening windows in a room with a coughing patient has no effect on the spread of TB (False)	138	69.0
31	If a fan is used in a room, opening windows will not provide additional benefits for TB infection control (False)	154	77.0
32	The windows in a room where there is a TB patient should not be opened because they have to be hidden from other people (False)	180	90.0
33	Presumptive TB cases in the waiting area should wait just as long as everyone else, and should not be rushed through the queue (False)	118	59.0

With regard to the frequency with which the nurses practised specific TBIC measures, Table 3 shows that less than 80% of the nurses answered “always” to any of the practice items. Opening of windows in the waiting area or room where a coughing patient is receiving attention was the most frequent measure practised “always” (76.5%), followed by instruction on cough etiquette (74.5%). Separation of coughing patients to a different waiting area was the measure least practised “always” (26.5%).

**Table 3:** Practice of TBIC measures by the respondents (*N* = 200)

SN	Practice item	Never (1) *n* (%)	Rarely (2) *n* (%)	Sometimes (3) *n* (%)	Often (4) *n* (%)	Always (5) *n* (%)
1	How frequently do you ask each patient when they enter the clinic, if they are coughing?	11 (5.5)	23 (11.5)	30 (15.0)	56 (28.0)	80 (40.0)
2	How frequently do you move coughing patients to wait at a nearby but separate waiting area?	15 (7.5)	43 (21.5)	52 (26.0)	37 (18.5)	53 (26.5)
3	How frequently do you instruct coughing patients to cover their mouth with tissues, handkerchiefs or their arm when coughing?	3 (1.5)	2 (1.0)	15 (7.5)	31 (15.5)	149 (74.5)
4	How frequently do you ensure that collection of sputum sample from a patient is done outdoors or in a separate, well-ventilated areas?	9 (4.5)	26 (13.0)	46 (23.0)	44 (22.0)	75 (37.5)
5	How frequently do you rapidly move a coughing patient to the front of the queue so he/she is attended to quickly to minimize the amount of time they spend in the clinic?	7 (3.5)	26 (13.0)	39 (19.5)	51 (25.5)	77 (38.5)
6	How frequently do you open windows in the patient waiting area or a room where a coughing patient is receiving attention (or check to see if they are open already)?	3 (1.5)	9 (4.5)	12 (6.0)	23 (11.5)	153 (76.5)

The nurses had mean scores of 68.2 and 79.9% on the knowledge and practice scales respectively, as depicted in Table 4. Using the cut-off of 80% to categorize the knowledge scores, the majority of them (89.5%) had poor knowledge scores. Likewise, the majority (94%) had poor practice scores based on the cut-off of 100%.

**Table 4:** TBIC knowledge and practice scores of the respondents (*N* = 200)

	Mean (SD), %	n (%)
*Score*		
Knowledge	68.2 (10.4)	
Practice	79.9 (15.3)	
*Score category*		
Knowledge		
Good		21 (10.5)
Poor		179 (89.5)
Practice		
Good		12 (6.0)
Poor		188 (94.0)

### Factors associated with TBIC-related knowledge and practices of the respondents

Bivariate logistic regression analysis revealed that there was no statistically significant association between the knowledge of the nurses and their socio-demographic characteristics (Table 5). Also, work experience was the only factor that was significantly associated with practices. The more experienced nurses (> 18 years of work experience) had lower odds of having good practices than the less experienced ones (OR 0.25, 95% CI 0.06–0.94). Furthermore, knowledge and practices were not significantly related, although the nurses with good knowledge had lower odds of obtaining good practice scores (OR 0.48, 95% CI 0–3.12).

**Table 5:** Association of socio-demographic characteristics of the respondents with their TBIC knowledge and practices (*N* = 200)

Exposure variable	Knowledge			Practices		
	OR	*p*-value	95% CI	OR	*p*-value	95% CI
Sex (Reference: Female)	1.74	0.62	0.19–15.6	3.33	0.29	0.36–31.0
Age (Reference: = < 44 years)	0.79	0.62	0.32–1.98	0.34	0.11	0.09–1.30
Experience category (Reference: = < 18 years)	1.08	0.87	0.43–2.69	0.25	0.04*	0.06–0.94
Professional rank (Reference: Senior)	1.35	0.52	0.55–3.35	0.46	0.26	0.12–1.75
Marital status (Reference: Married)	1.15	0.86	0.24–5.42	0.98	0.98	0.12–8.06
Knowledge (Reference: Poor)				0.48^†^	0.51^†^	0.00–3.12^†^

*p < 0.05; ^†^Exact logistic regression

## Discussion

This study was conducted to assess the TBIC-related knowledge and practices of nurses in two secondary health facilities in Ibadan, Nigeria, and their association with the socio-demographic characteristics of the nurses. The results revealed mean knowledge and practice scores of 68.2 and 79.9% respectively. Furthermore, with the cut-off for good knowledge and practice scores set at 80 and 100% respectively, it was observed that the majority of them had poor knowledge and practices. This is consistent with reports from other studies in Nigeria where, generally, poor levels have been demonstrated among HCWs [18, 19, 23, 24].

Various methods of assessing and scoring TB-related knowledge and practices have been observed from previous studies. While some of these simply stated the mean and used this as the cut-off to categorize the scores of the participants, others had arbitrary cut-off points [25, 27]. Additionally, the categories may be varied: (i) good, moderate, and poor; (ii) good and poor; (iii) good, fair, and poor; and (iv) proper, and improper [7, 15, 27, 28]. The majority of the nurses in this study (> 80%) were able to correctly identify the constitutional symptoms of TB (cough of 2–3 weeks duration, bloody sputum, night sweats weight loss and fever). This is similar to the findings by Bhebhe et al. among HCWs in Lesotho, except that in their study, only 53.5% considered fever to be a symptom of TB [7]. Most of the nurses gave correct responses to the questions related to the mode of transmission of TB. For instance, 95% recognized that TB can be transmitted by coughing (96.5%), and its transmission can be reduced by practising cough etiquette/hygiene (96%), and by opening the windows of a room that has a TB patient in it (90%). This agrees with the findings in studies conducted in Nigeria and Northwest Ethiopia [24, 28]. It also aligns with a South African study, where Kanjee et al. reported that “most of the information (knowledge) items were answered correctly by over 70% of respondents with some exceptions” and that the “HCWs were generally well informed about TB transmission” [13]. Other researchers have made similar observations among HCWs in Free State Province, South Africa [15].

The mean knowledge score of 68.2% reported in this study is higher than the findings of 61, and 61.5% reported by previous investigators [9, 33]. The poor knowledge level of TBIC conforms with the results of a study by Woith et al. among HCWs in Russia [34]. In contrast to the poor knowledge noticed in the present study, some researchers have previously reported “good” or “adequate” TBIC knowledge among HCWs, although lower cut-off points were used in their studies. For instance, Bhebhe et al. reported that 89.2% of HCWs in their study in Lesotho had “appropriate” TBIC knowledge, but the cut-off used to define “good” was 70%, which is lower than 80% adopted for the present study [7]. Furthermore, the mean score of 61.5% reported by them is lower than 68.2% observed in this study. Buregyeya et al. similarly reported that 69% of HCWs in a study in Uganda had adequate TBIC knowledge, with the cut-off taken as 70% [27]. A study in Ethiopia by Temesgen and Demissie revealed that 74.4% of health professionals had “good” knowledge, using a cut-off of 60% [26].

In terms of the practice of TBIC measures, only two out of the six items had more than 70% of the nurses reporting them as “always” practised: cough etiquette/hygiene and opening of windows. Ekuma et al. also reported similar poor “always” results for practice items in a study in Nigeria [24]. This is, however, different from the reports of a South African study by Engelbrecht et al., where four items out of 12 had more than 80% of the respondents who “always” practised them: patient fast-tracking, screening, window-opening, and collection of sputum specimens from coughing patients [15]. However, it is notable that the frequency of practice of the measures in that study, just like in the present study, was self-reported by the respondents; and the researchers noted a discrepancy between the self-reports and observed practices. We noticed in our study that the proportions of nurses reporting various TBIC measures as “always” practised were less than those that recorded correct responses to related questions on the knowledge scale. This incongruity suggests that, although good levels of nurses’ TBIC knowledge have been shown to be closely associated with good practices, it is not its only determinant [15]. Other factors that influence proper TBIC practices include clear policy directives, appropriate triage system and separation of coughing patients, availability of personal protection equipment, reasonable workload, adequate and well-ventilated clinic space**,** among others [35]. Findings from studies conducted in LMIC, where cost-effective TBIC measures are best suited, are in overwhelming support of the results of the present study. Inadequate practice of TBIC measures have been reported in Nigeria, South Africa, Lesotho and Ethiopia [8, 9, 15, 28]. Tamir and his co-workers, using 80% as their cut-off, found that only 38% of HCWs in their Ethiopian study had overall proper TBIC practices [28]. Even though Temesgen and Demissie reported an overall “good” TBIC practice in Ethiopia, with a cut-off of 50%, specific practices were still poor [26]. Poor levels of implementation of TBIC measures were also reported by Bhebhe et al. and Kanjee et al. in Lesotho and South Africa respectively [7, 13] The divergence in the proportions with good knowledge and practices noticed between the present study and previous ones cited could be due to the different cut-off points and scoring systems used. It is noteworthy that higher cut-off points were used in the present study. However, the finding of large proportions of nurses with poor levels of knowledge and practices in this study is not completely unexpected because, although TBIC guidelines had been released in the country at the time the study was conducted, implementation was still in its infancy. Moreover, many facilities, including the study sites, were yet to benefit from the roll-out package [14, 19, 20, 23].

The nurses’ TBIC knowledge was not significantly related to all the socio-demographic factors considered. This result is in conformity with the observations from previous studies. Temesgen and Demissie reported that TBIC knowledge among HCWs was not significantly associated with work experience and age category [26]. Buregyeya et al. also noted that it was not associated with age and sex. On the contrary, Gizaw et al. showed an association between knowledge and work experience in a study in Ethiopia, with HCWs who had more than 6 years’ work experience being more knowledgeable than those with less than 3 years’ experience [25, 27]. The lack of association they observed between knowledge and age, however, agrees with our findings. Besides the revelation from the present study that the more experienced nurses had statistically significant lower odds of having good practices, the observations concerning the other socio-demographic factors are in alignment with some previous studies. According to Mugomeri et al., the nurses’ age and TB ward work experience did not significantly influence their practice of TBIC measures in a study in Lesotho [9]. Similarly, Temesgen and Demissie noted that the practices were not related to work experience and age category [26]. Also, work experience, age, gender and marital status were not statistically related to practice in the study by Tamir et al. [28]. The more experienced nurses in the present study were less likely to obtain good practices maybe because most of them, who are usually in the senior cadre, serve as unit heads or managers and their work duties include mainly managerial functions such as managing work schedule and roster, ensuring compliance with work policies and protocols, commodity management, among others. They may not necessarily carry out actual TBIC work practices, in line with official responsibilities assigned to the different professional levels. Some investigators in Nigeria have reported that nurse managers tend to focus more on their managerial duties at the expense of their clinical roles [36, 37]. The distribution of the respondents in terms of sex and marital status was heavily skewed in favour of females (97.0%) and married ones (91.5%). This pattern reflects the profile of the nursing workforce in Nigeria, as reported by other investigators [38]. Furthermore, the wide confidence intervals recorded for the regression analysis using the two factors as separate independent variables, and knowledge and practice as dependent variables indicate low precision. These elements should be considered when interpreting the results related to sex and marital status.

The association between TBIC knowledge and practices was not statistically significant, although the nurses with good knowledge scores were less likely to obtain good practice scores. This should, however, be interpreted with caution as the 2 × 2 contingency table showed that there were no nurses with good knowledge scores that also had good practice scores, hence the use of exact logistic regression approach to produce the point estimate and confidence interval, in line with the recommendations by Hosmer and Lemeshow for analyzing cells with zero or sparse counts [39]. This absence of association between knowledge and practices aligns with the findings by Gizaw et al. [25]. It, however, contracts with reports from South Africa and Northwest Ethiopia, where a significant association was reported between them [15, 26]. Furthermore, TBIC knowledge was reported to be significantly associated with training on TBIC in some of the previous studies referenced here [25–27]. Knowledge might not have significantly influenced practices in the present study because at the time the study was conducted, nurses at the study sites were yet to be trained on the newly-introduced TBIC package [14, 23]. The findings from this study suggest that diffusion of actions and professional socialization might have influenced the routine work practices of the nurses, including performance of TB preventive measures, irrespective of their knowledge and socio-demographic characteristics as these were generally not significantly associated with their TBIC practices [40, 41]. However, the positive influence of TBIC training on specific aspects or overall practice of TB preventive measures revealed in some studies, and the well-known interplay of training and knowledge on practice underscore the importance of conducting trainings on TBIC for the nurses in order to equip them with essential skills to improve their practices [25, 26, 42].

### Limitations of the study

It was difficult to carry out direct observation of TBIC practices by the nurses as this is time-consuming and requires the engagement of more research personnel as observers. Self-reports were relied on and there is the likelihood of a discrepancy between this and direct observation of actual practices, as previously reported by Engelbrecht et al. [15]. There could have been over-reporting of the practices by the participants because of the social acceptability of being perceived as doing the proper thing (social desirability bias). Also, for the purpose of obtaining an all-inclusive sample, nurses in all the units of the hospitals (out-patient units, in-patient wards, emergency room, operating theatre, among others) were encouraged to participate in the study. Although the nurses rotate through various work units, some of the duties and assigned tasks may not be directly related to TB care and it might have been challenging for participants who had not recently been involved in providing TB-related care to respond correctly to the practice items (recall bias). Furthermore, the study questionnaire tended towards administrative and environmental control measures (specifically, natural ventilation system) as managerial control measures are more in the purview of the facility management while personal protection equipment (respirators) are mostly available for use in specialized MDR-TB centres. It would be beneficial to further review and expand the items in the study instrument to make for the implementation of a more comprehensive package of TBIC measures.

## Conclusions

This cross-sectional study revealed small proportions of nurses with good TBIC-related knowledge and practices in two secondary health facilities in Ibadan, Nigeria. It also shows that the nurses’ socio-demographic characteristics were not significantly related to their knowledge and practices, except for the association between experience and practices. This study has provided baseline information that can be used by policy makers at various levels to design interventions aimed at improving the TBIC knowledge and practices of nurses and other HCWs. It can also be used to benchmark the evaluation of TBIC interventions, especially in settings where TBIC guidelines have not been optimally implemented. It is recommended that training on TBIC should be provided to nurses. This will equip them with the knowledge and skills vital for adequate implementation of TB preventive measures. In addition, health facility managers should ensure the dissemination of clear policy directives and the establishment of an appropriate monitoring and supervision system for TBIC.

## Supplementary information

**Additional file 1.** Study Questionnaire.

## Data Availability

The dataset used and analyzed during the current study is available from the corresponding author on reasonable request.

## Abbreviations

CDC: U.S. Centers for Disease Control and Prevention
CI: Confidence interval
HBC: High burden countries
HCW: Healthcare worker
HIV: Human Immunodeficiency Virus
LGA: Local Government Area
LMIC: Low- and medium-income countries
MDR-TB: Multi-drug resistant tuberculosis
MTB: *Mycobacterium tuberculosis*
OR: Odds Ratio
PPE: Personal protective equipment
PTB: Pulmonary tuberculosis
RR-TB: Rifampicin-resistant tuberculosis
TB: Tuberculosis
TBIC: Tuberculosis infection control
WHO: World Health Organization
